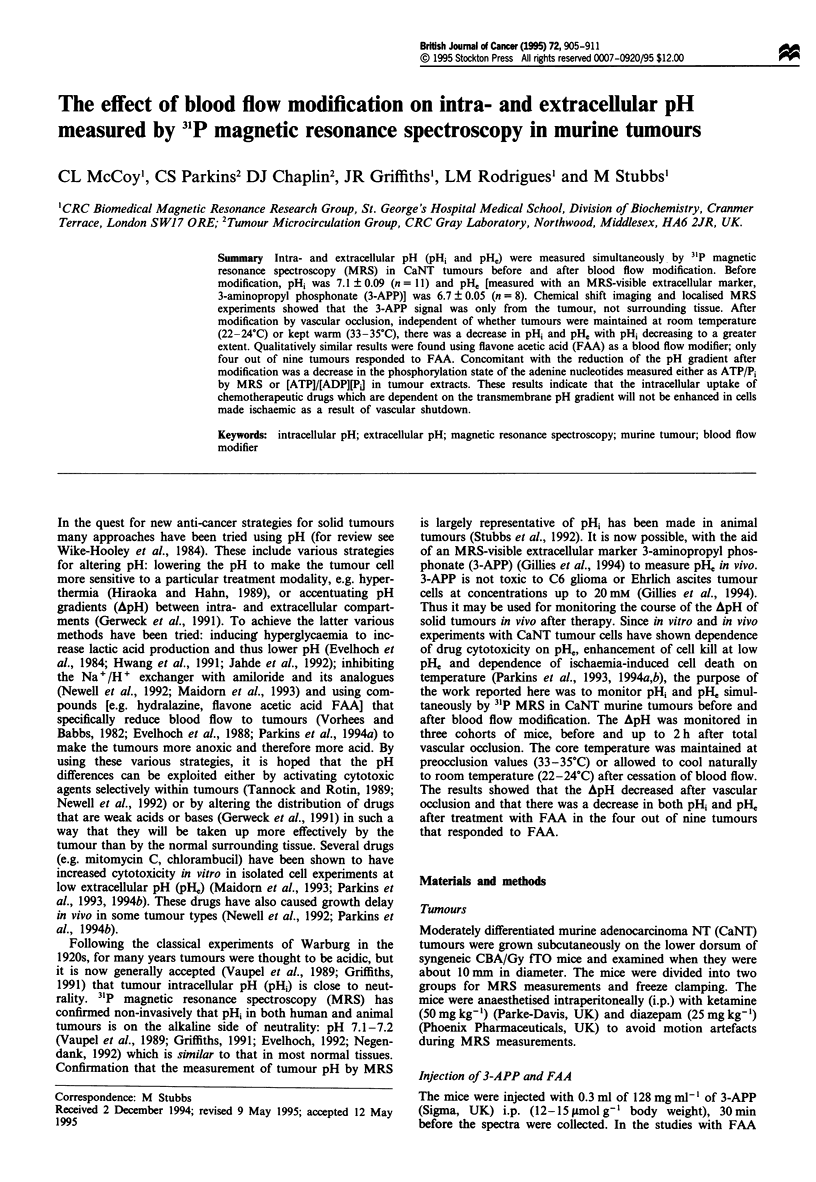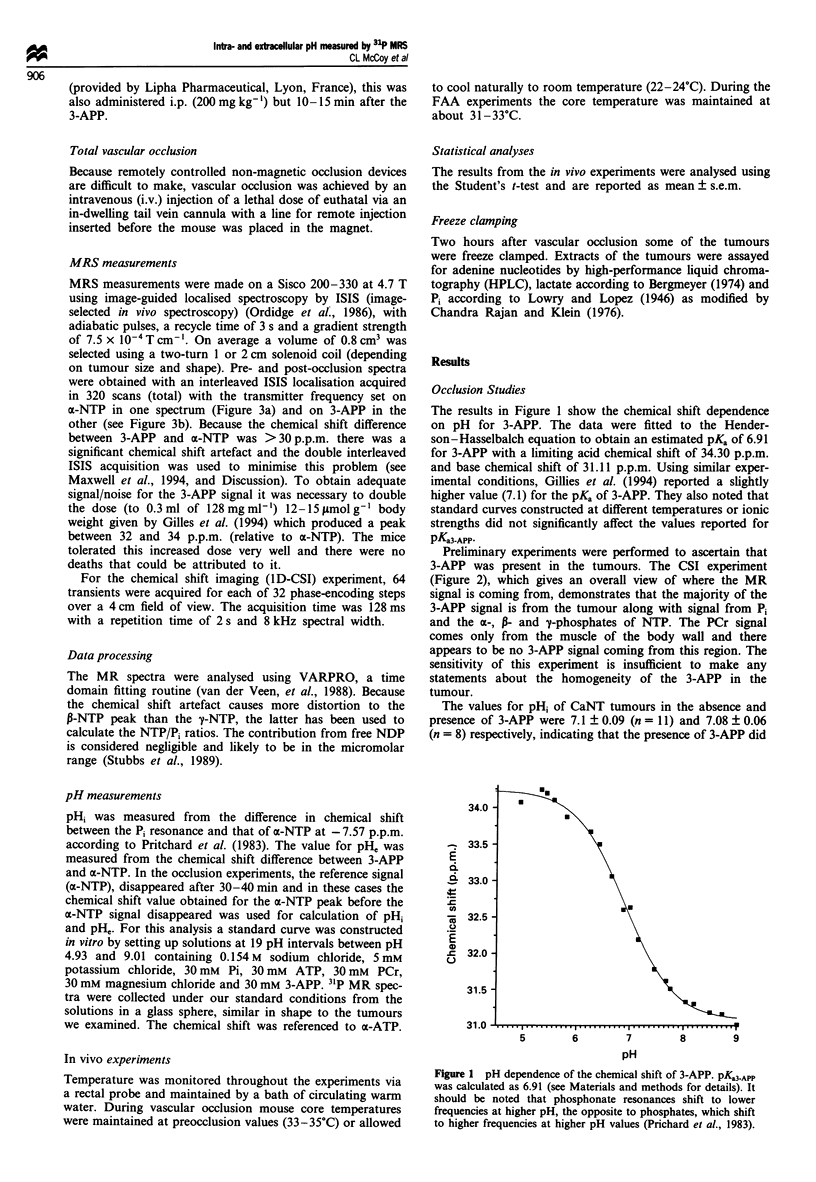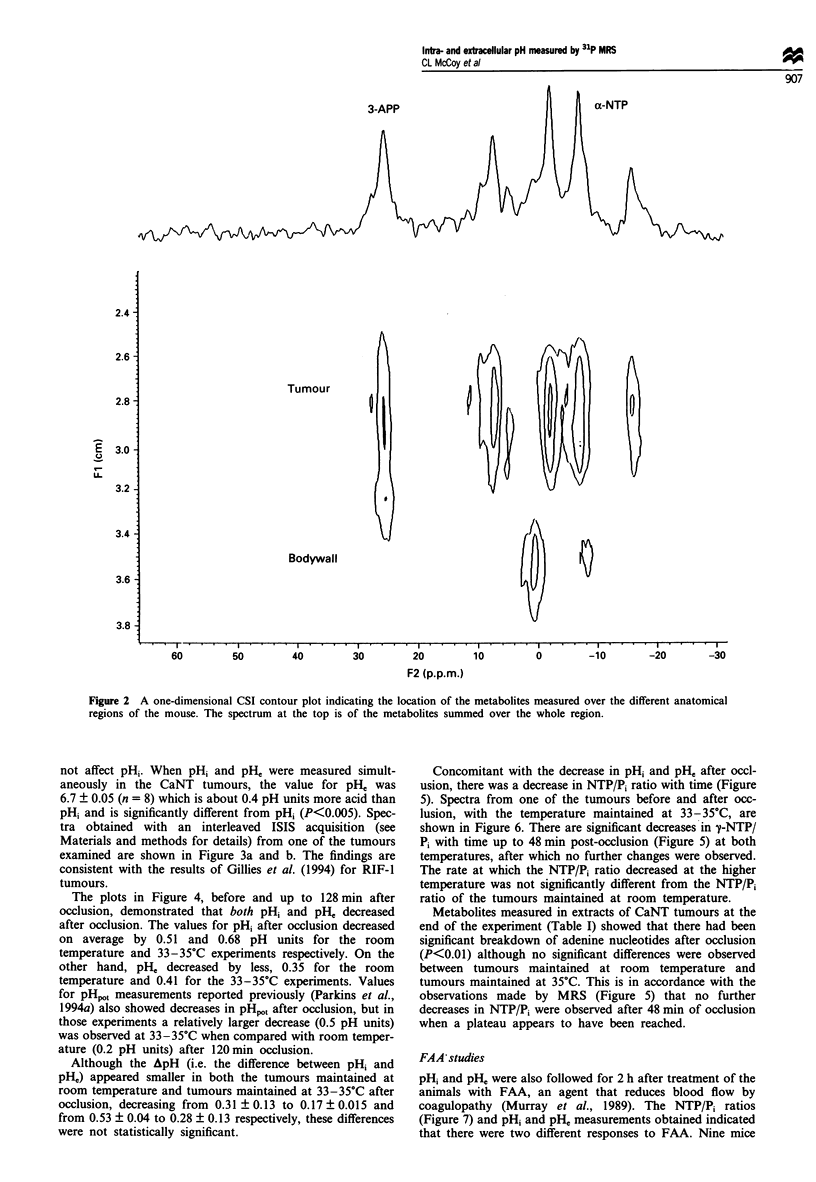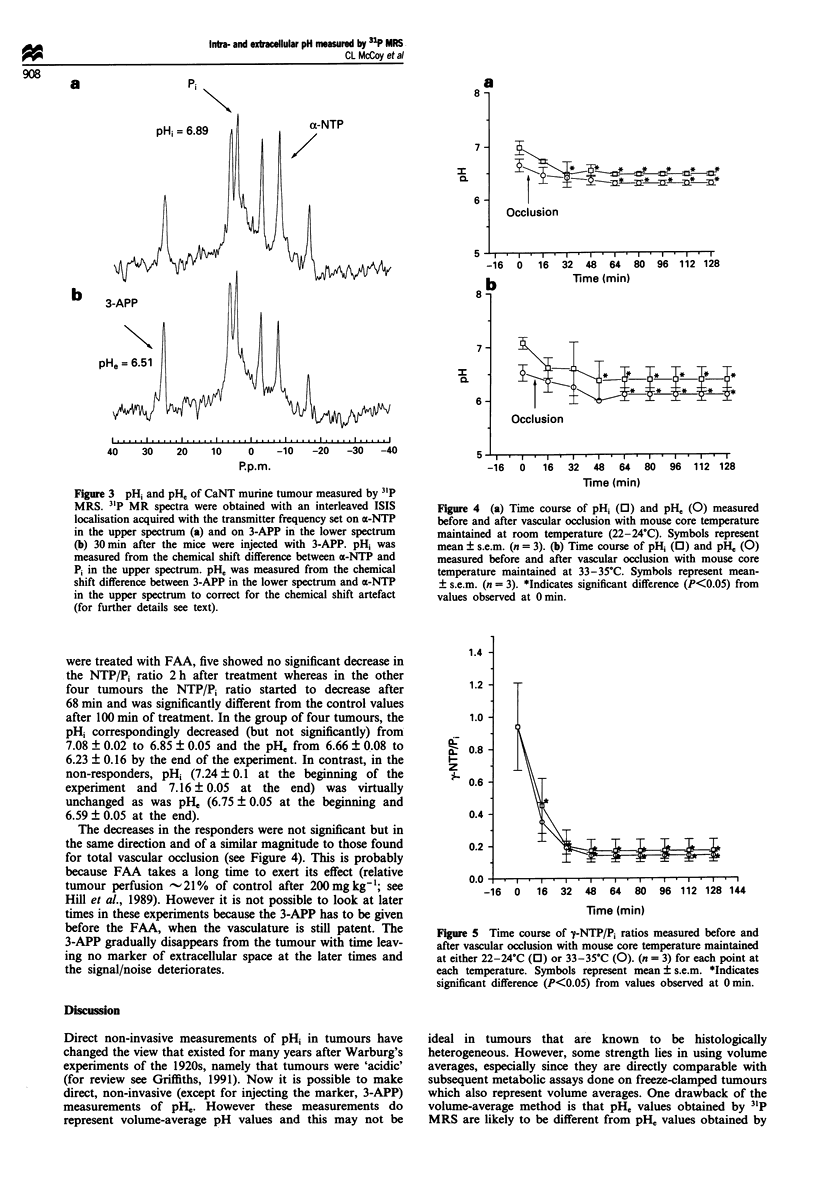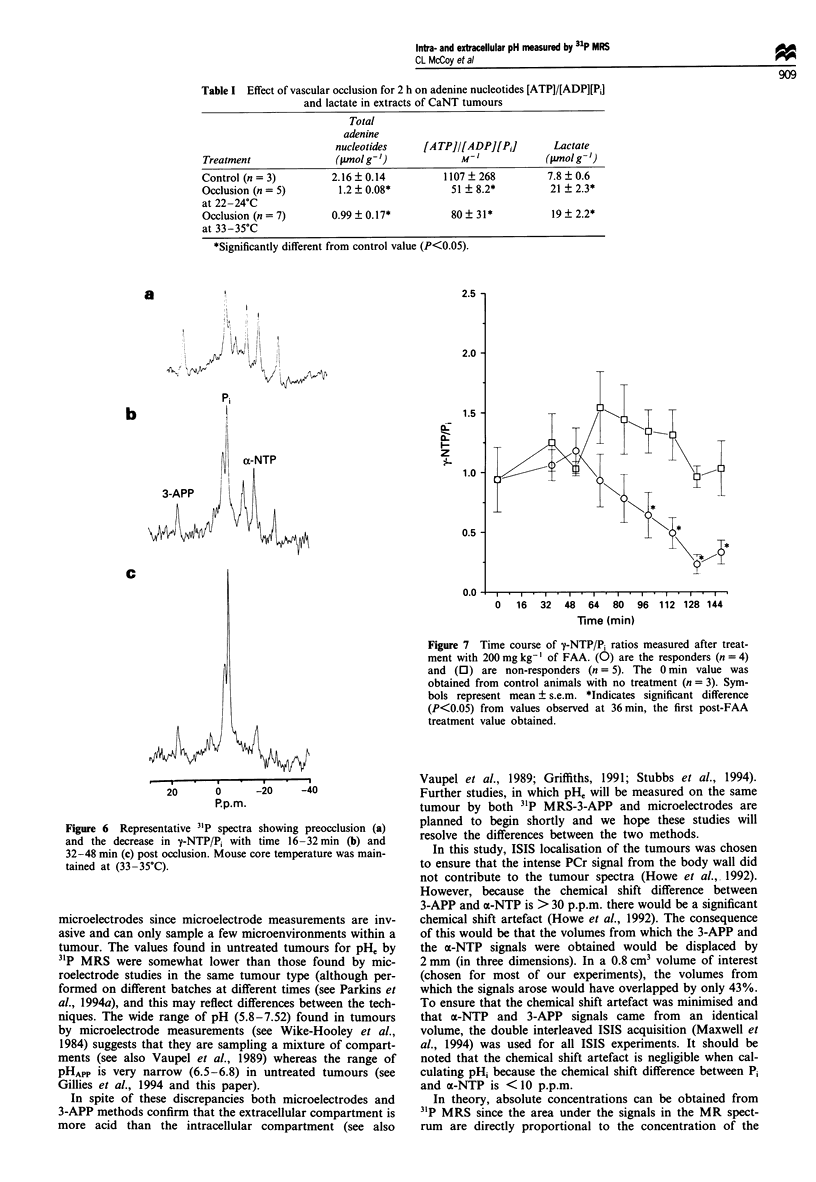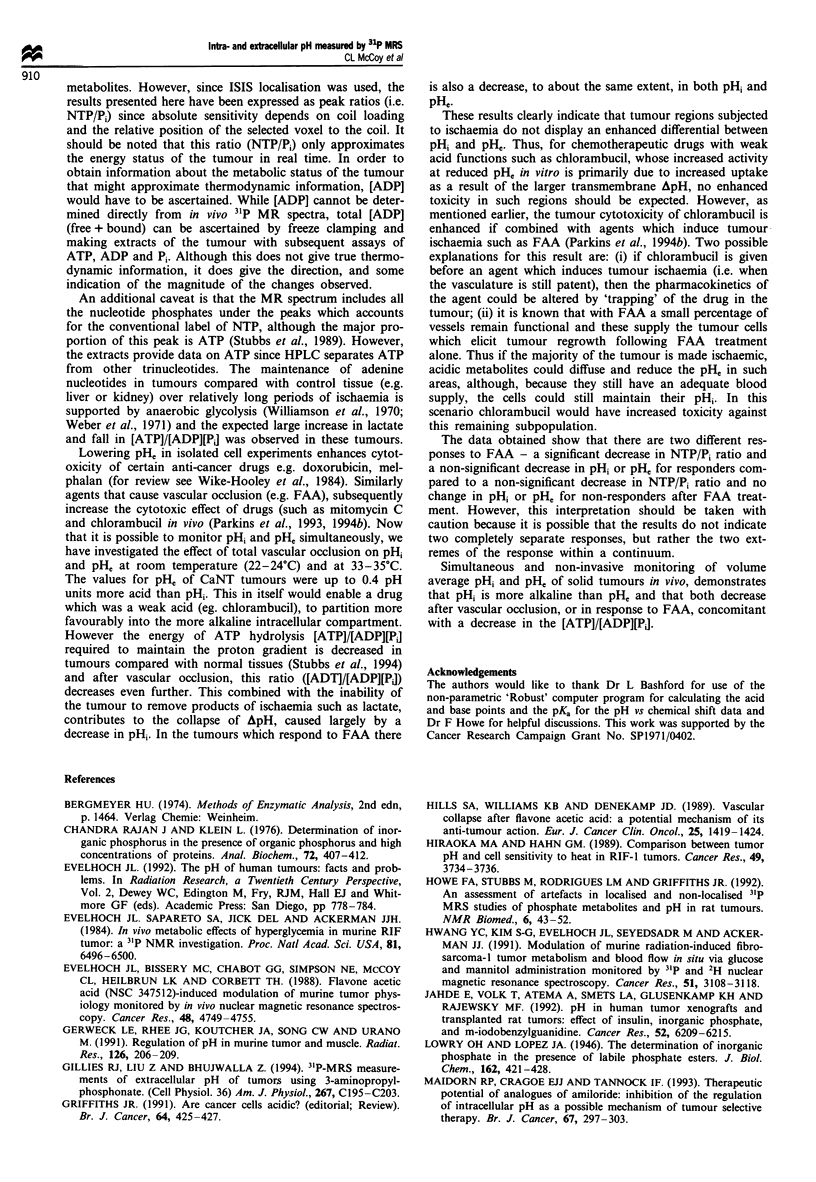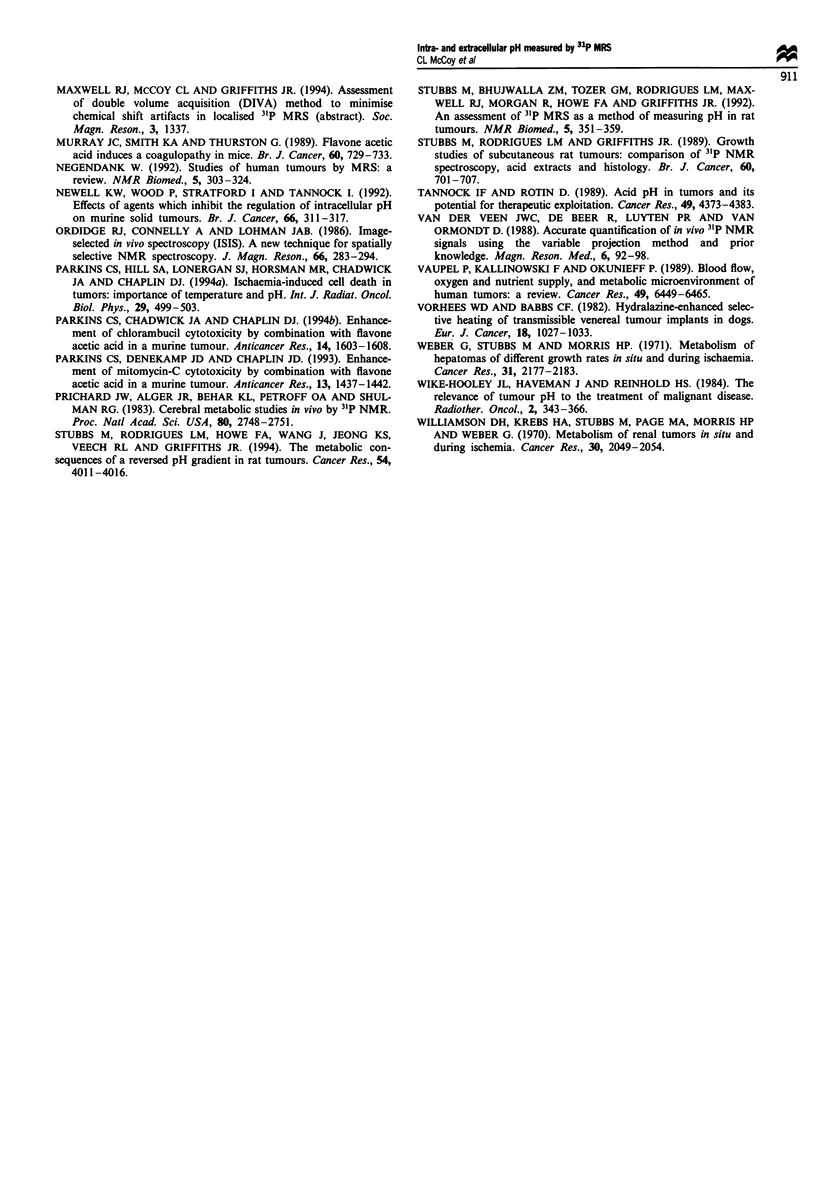# The effect of blood flow modification on intra- and extracellular pH measured by 31P magnetic resonance spectroscopy in murine tumours.

**DOI:** 10.1038/bjc.1995.431

**Published:** 1995-10

**Authors:** C. L. McCoy, C. S. Parkins, D. J. Chaplin, J. R. Griffiths, L. M. Rodrigues, M. Stubbs

**Affiliations:** CRC Biomedical Magnetic Resonance Research Group, St. George's Hospital Medical School, London, UK.

## Abstract

Intra- and extracellular pH (pHi and pHe) were measured simultaneously by 31P magnetic resonance spectroscopy (MRS) in CaNT tumours before and after blood flow modification. Before modification, pHi was 7.1 +/- 0.09 (n = 11) and pHe [measured with an MRS-visible extracellular marker, 3-aminopropyl phosphonate (3-APP)] was 6.7 +/- 0.05 (n = 8). Chemical shift imaging and localised MRS experiments showed that the 3-APP signal was only from the tumour, not surrounding tissue. After modification by vascular occlusion, independent of whether tumours were maintained at room temperature (22-24 degrees C) or kept warm (33-35 degrees C), there was a decrease in pHi and pHe with pHi decreasing to a greater extent. Qualitatively similar results were found using flavone acetic acid (FAA) as a blood flow modifier; only four out of nine tumours responded to FAA. Concomitant with the reduction of the pH gradient after modification was a decrease in the phosphorylation state of the adenine nucleotides measured either as ATP/Pi by MRS or [ATP]/[ADP][P(i)] in tumour extracts. These results indicate that the intracellular uptake of chemotherapeutic drugs which are dependent on the transmembrane pH gradient will not be enhanced in cells made ischaemic as a result of vascular shutdown.


British Journal of Cancer (1995) 72, 905-911
© 1995 Stockton Press All rights reserved 0007-0920/95 $12.00

### The effect of blood flow modification on intra- and extracellular pH measured by $^{31}P$ magnetic resonance spectroscopy in murine tumours

CL McCoy[1], CS Parkins[2] DJ Chaplin[2], JR Griffiths[1], LM Rodrigues[1] and M Stubbs[1]

[1]*CRC Biomedical Magnetic Resonance Research Group, St. George's Hospital Medical School, Division of Biochemistry, Cranmer Terrace, London SW17 0RE;* [2]*Tumour Microcirculation Group, CRC Gray Laboratory, Northwood, Middlesex, HA6 2JR, UK.*

**Summary** Intra- and extracellular pH ($pH_i$ and $pH_e$) were measured simultaneously by $^{31}P$ magnetic resonance spectroscopy (MRS) in CaNT tumours before and after blood flow modification. Before modification, $pH_i$ was $7.1 \pm 0.09$ ($n = 11$) and $pH_e$ [measured with an MRS-visible extracellular marker, 3-aminopropyl phosphonate (3-APP)] was $6.7 \pm 0.05$ ($n = 8$). Chemical shift imaging and localised MRS experiments showed that the 3-APP signal was only from the tumour, not surrounding tissue. After modification by vascular occlusion, independent of whether tumours were maintained at room temperature (22–24°C) or kept warm (33–35°C), there was a decrease in $pH_i$ and $pH_e$ with $pH_i$ decreasing to a greater extent. Qualitatively similar results were found using flavone acetic acid (FAA) as a blood flow modifier; only four out of nine tumours responded to FAA. Concomitant with the reduction of the pH gradient after modification was a decrease in the phosphorylation state of the adenine nucleotides measured either as $ATP/P_i$ by MRS or $[ATP]/[ADP][P_i]$ in tumour extracts. These results indicate that the intracellular uptake of chemotherapeutic drugs which are dependent on the transmembrane pH gradient will not be enhanced in cells made ischaemic as a result of vascular shutdown.

**Keywords:** intracellular pH; extracellular pH; magnetic resonance spectroscopy; murine tumour; blood flow modifier

In the quest for new anti-cancer strategies for solid tumours many approaches have been tried using pH (for review see Wike-Hooley *et al.*, 1984). These include various strategies for altering pH: lowering the pH to make the tumour cell more sensitive to a particular treatment modality, e.g. hyperthermia (Hiraoka and Hahn, 1989), or accentuating pH gradients (ΔpH) between intra- and extracellular compartments (Gerweck *et al.*, 1991). To achieve the latter various methods have been tried: inducing hyperglycaemia to increase lactic acid production and thus lower pH (Evelhoch *et al.*, 1984; Hwang *et al.*, 1991; Jahde *et al.*, 1992); inhibiting the $Na^+/H^+$ exchanger with amiloride and its analogues (Newell *et al.*, 1992; Maidorn *et al.*, 1993) and using compounds [e.g. hydralazine, flavone acetic acid FAA] that specifically reduce blood flow to tumours (Vorhees and Babbs, 1982; Evelhoch *et al.*, 1988; Parkins *et al.*, 1994*a*) to make the tumours more anoxic and therefore more acid. By using these various strategies, it is hoped that the pH differences can be exploited either by activating cytotoxic agents selectively within tumours (Tannock and Rotin, 1989; Newell *et al.*, 1992) or by altering the distribution of drugs that are weak acids or bases (Gerweck *et al.*, 1991) in such a way that they will be taken up more effectively by the tumour than by the normal surrounding tissue. Several drugs (e.g. mitomycin C, chlorambucil) have been shown to have increased cytotoxicity *in vitro* in isolated cell experiments at low extracellular pH ($pH_e$) (Maidorn *et al.*, 1993; Parkins *et al.*, 1993, 1994*b*). These drugs have also caused growth delay *in vivo* in some tumour types (Newell *et al.*, 1992; Parkins *et al.*, 1994*b*).

Following the classical experiments of Warburg in the 1920s, for many years tumours were thought to be acidic, but it is now generally accepted (Vaupel *et al.*, 1989; Griffiths, 1991) that tumour intracellular pH ($pH_i$) is close to neutrality. $^{31}P$ magnetic resonance spectroscopy (MRS) has confirmed non-invasively that $pH_i$ in both human and animal tumours is on the alkaline side of neutrality: pH 7.1–7.2 (Vaupel *et al.*, 1989; Griffiths, 1991; Evelhoch, 1992; Negendank, 1992) which is *similar* to that in most normal tissues. Confirmation that the measurement of tumour pH by MRS is largely representative of $pH_i$ has been made in animal tumours (Stubbs *et al.*, 1992). It is now possible, with the aid of an MRS-visible extracellular marker 3-aminopropyl phosphonate (3-APP) (Gillies *et al.*, 1994) to measure $pH_e$ *in vivo*. 3-APP is not toxic to C6 glioma or Ehrlich ascites tumour cells at concentrations up to 20 mM (Gillies *et al.*, 1994). Thus it may be used for monitoring the course of the ΔpH of solid tumours *in vivo* after therapy. Since *in vitro* and *in vivo* experiments with CaNT tumour cells have shown dependence of drug cytotoxicity on $pH_e$, enhancement of cell kill at low $pH_e$ and dependence of ischaemia-induced cell death on temperature (Parkins *et al.*, 1993, 1994*a*,*b*), the purpose of the work reported here was to monitor $pH_i$ and $pH_e$ simultaneously by $^{31}P$ MRS in CaNT murine tumours before and after blood flow modification. The ΔpH was monitored in three cohorts of mice, before and up to 2 h after total vascular occlusion. The core temperature was maintained at preocclusion values (33–35°C) or allowed to cool naturally to room temperature (22–24°C) after cessation of blood flow. The results showed that the ΔpH decreased after vascular occlusion and that there was a decrease in both $pH_i$ and $pH_e$ after treatment with FAA in the four out of nine tumours that responded to FAA.

### Materials and methods

### *Tumours*

Moderately differentiated murine adenocarcinoma NT (CaNT) tumours were grown subcutaneously on the lower dorsum of syngeneic CBA/Gy fTO mice and examined when they were about 10 mm in diameter. The mice were divided into two groups for MRS measurements and freeze clamping. The mice were anaesthetised intraperitoneally (i.p.) with ketamine ($50\ mg\ kg^{-1}$) (Parke-Davis, UK) and diazepam ($25\ mg\ kg^{-1}$) (Phoenix Pharmaceuticals, UK) to avoid motion artefacts during MRS measurements.

### *Injection of 3-APP and FAA*

The mice were injected with 0.3 ml of $128\ mg\ ml^{-1}$ of 3-APP (Sigma, UK) i.p. ($12-15\ \mu mol\ g^{-1}$ body weight), 30 min before the spectra were collected. In the studies with FAA

Correspondence: M Stubbs
Received 2 December 1994; revised 9 May 1995; accepted 12 May 1995

(provided by Lipha Pharmaceutical, Lyon, France), this was also administered i.p. (200 mg $kg^{-1}$) but 10–15 min after the 3-APP.

*Total vascular occlusion*

Because remotely controlled non-magnetic occlusion devices are difficult to make, vascular occlusion was achieved by an intravenous (i.v.) injection of a lethal dose of euthatal via an in-dwelling tail vein cannula with a line for remote injection inserted before the mouse was placed in the magnet.

*MRS measurements*

MRS measurements were made on a Sisco 200–330 at 4.7 T using image-guided localised spectroscopy by ISIS (image-selected *in vivo* spectroscopy) (Ordidge *et al.*, 1986), with adiabatic pulses, a recycle time of 3 s and a gradient strength of $7.5 \times 10^{-4}$ T $cm^{-1}$. On average a volume of 0.8 $cm^3$ was selected using a two-turn 1 or 2 cm solenoid coil (depending on tumour size and shape). Pre- and post-occlusion spectra were obtained with an interleaved ISIS localisation acquired in 320 scans (total) with the transmitter frequency set on α-NTP in one spectrum (Figure 3a) and on 3-APP in the other (see Figure 3b). Because the chemical shift difference between 3-APP and α-NTP was >30 p.p.m. there was a significant chemical shift artefact and the double interleaved ISIS acquisition was used to minimise this problem (see Maxwell *et al.*, 1994, and Discussion). To obtain adequate signal/noise for the 3-APP signal it was necessary to double the dose (to 0.3 ml of 128 mg $ml^{-1}$) 12–15 μmol $g^{-1}$ body weight given by Gilles *et al.* (1994) which produced a peak between 32 and 34 p.p.m. (relative to α-NTP). The mice tolerated this increased dose very well and there were no deaths that could be attributed to it.

For the chemical shift imaging (1D-CSI) experiment, 64 transients were acquired for each of 32 phase-encoding steps over a 4 cm field of view. The acquisition time was 128 ms with a repetition time of 2 s and 8 kHz spectral width.

*Data processing*

The MR spectra were analysed using VARPRO, a time domain fitting routine (van der Veen, *et al.*, 1988). Because the chemical shift artefact causes more distortion to the β-NTP peak than the γ-NTP, the latter has been used to calculate the NTP/$P_i$ ratios. The contribution from free NDP is considered negligible and likely to be in the micromolar range (Stubbs *et al.*, 1989).

*pH measurements*

$pH_i$ was measured from the difference in chemical shift between the $P_i$ resonance and that of α-NTP at −7.57 p.p.m. according to Pritchard *et al.* (1983). The value for $pH_e$ was measured from the chemical shift difference between 3-APP and α-NTP. In the occlusion experiments, the reference signal (α-NTP), disappeared after 30–40 min and in these cases the chemical shift value obtained for the α-NTP peak before the α-NTP signal disappeared was used for calculation of $pH_i$ and $pH_e$. For this analysis a standard curve was constructed *in vitro* by setting up solutions at 19 pH intervals between pH 4.93 and 9.01 containing 0.154 M sodium chloride, 5 mM potassium chloride, 30 mM Pi, 30 mM ATP, 30 mM PCr, 30 mM magnesium chloride and 30 mM 3-APP. $^{31}$P MR spectra were collected under our standard conditions from the solutions in a glass sphere, similar in shape to the tumours we examined. The chemical shift was referenced to α-ATP.

In vivo *experiments*

Temperature was monitored throughout the experiments via a rectal probe and maintained by a bath of circulating warm water. During vascular occlusion mouse core temperatures were maintained at preocclusion values (33–35°C) or allowed to cool naturally to room temperature (22–24°C). During the FAA experiments the core temperature was maintained at about 31–33°C.

*Statistical analyses*

The results from the *in vivo* experiments were analysed using the Student's *t*-test and are reported as mean ± s.e.m.

*Freeze clamping*

Two hours after vascular occlusion some of the tumours were freeze clamped. Extracts of the tumours were assayed for adenine nucleotides by high-performance liquid chromatography (HPLC), lactate according to Bergmeyer (1974) and $P_i$ according to Lowry and Lopez (1946) as modified by Chandra Rajan and Klein (1976).

### Results

*Occlusion Studies*

The results in Figure 1 show the chemical shift dependence on pH for 3-APP. The data were fitted to the Henderson–Hasselbalch equation to obtain an estimated $pK_a$ of 6.91 for 3-APP with a limiting acid chemical shift of 34.30 p.p.m. and base chemical shift of 31.11 p.p.m. Using similar experimental conditions, Gillies *et al.* (1994) reported a slightly higher value (7.1) for the $pK_a$ of 3-APP. They also noted that standard curves constructed at different temperatures or ionic strengths did not significantly affect the values reported for $pK_{a3\text{-}APP}$.

Preliminary experiments were performed to ascertain that 3-APP was present in the tumours. The CSI experiment (Figure 2), which gives an overall view of where the MR signal is coming from, demonstrates that the majority of the 3-APP signal is from the tumour along with signal from $P_i$ and the α-, β- and γ-phosphates of NTP. The PCr signal comes only from the muscle of the body wall and there appears to be no 3-APP signal coming from this region. The sensitivity of this experiment is insufficient to make any statements about the homogeneity of the 3-APP in the tumour.

The values for $pH_i$ of CaNT tumours in the absence and presence of 3-APP were 7.1 ± 0.09 ($n = 11$) and 7.08 ± 0.06 ($n = 8$) respectively, indicating that the presence of 3-APP did

**Figure 1** pH dependence of the chemical shift of 3-APP. $pK_{a3\text{-}APP}$ was calculated as 6.91 (see Materials and methods for details). It should be noted that phosphonate resonances shift to lower frequencies at higher pH, the opposite to phosphates, which shift to higher frequencies at higher pH values (Prichard *et al.*, 1983).

**Figure 2** A one-dimensional CSI contour plot indicating the location of the metabolites measured over the different anatomical regions of the mouse. The spectrum at the top is of the metabolites summed over the whole region.

not affect $pH_i$. When $pH_i$ and $pH_e$ were measured simultaneously in the CaNT tumours, the value for $pH_e$ was $6.7 \pm 0.05$ ($n = 8$) which is about 0.4 pH units more acid than $pH_i$ and is significantly different from $pH_i$ ($P<0.005$). Spectra obtained with an interleaved ISIS acquisition (see Materials and methods for details) from one of the tumours examined are shown in Figure 3a and b. The findings are consistent with the results of Gillies *et al.* (1994) for RIF-1 tumours.

The plots in Figure 4, before and up to 128 min after occlusion, demonstrated that *both* $pH_i$ and $pH_e$ decreased after occlusion. The values for $pH_i$ after occlusion decreased on average by 0.51 and 0.68 pH units for the room temperature and 33–35°C experiments respectively. On the other hand, $pH_e$ decreased by less, 0.35 for the room temperature and 0.41 for the 33–35°C experiments. Values for $pH_{pot}$ measurements reported previously (Parkins *et al.*, 1994*a*) also showed decreases in $pH_{pot}$ after occlusion, but in those experiments a relatively larger decrease (0.5 pH units) was observed at 33–35°C when compared with room temperature (0.2 pH units) after 120 min occlusion.

Although the ΔpH (i.e. the difference between $pH_i$ and $pH_e$) appeared smaller in both the tumours maintained at room temperature and tumours maintained at 33–35°C after occlusion, decreasing from $0.31 \pm 0.13$ to $0.17 \pm 0.015$ and from $0.53 \pm 0.04$ to $0.28 \pm 0.13$ respectively, these differences were not statistically significant.

Concomitant with the decrease in $pH_i$ and $pH_e$ after occlusion, there was a decrease in NTP/$P_i$ ratio with time (Figure 5). Spectra from one of the tumours before and after occlusion, with the temperature maintained at 33–35°C, are shown in Figure 6. There are significant decreases in γ-NTP/$P_i$ with time up to 48 min post-occlusion (Figure 5) at both temperatures, after which no further changes were observed. The rate at which the NTP/$P_i$ ratio decreased at the higher temperature was not significantly different from the NTP/$P_i$ ratio of the tumours maintained at room temperature.

Metabolites measured in extracts of CaNT tumours at the end of the experiment (Table I) showed that there had been significant breakdown of adenine nucleotides after occlusion ($P<0.01$) although no significant differences were observed between tumours maintained at room temperature and tumours maintained at 35°C. This is in accordance with the observations made by MRS (Figure 5) that no further decreases in NTP/$P_i$ were observed after 48 min of occlusion when a plateau appears to have been reached.

### *FAA studies*

$pH_i$ and $pH_e$ were also followed for 2 h after treatment of the animals with FAA, an agent that reduces blood flow by coagulopathy (Murray *et al.*, 1989). The NTP/$P_i$ ratios (Figure 7) and $pH_i$ and $pH_e$ measurements obtained indicated that there were two different responses to FAA. Nine mice

**Figure 3** $pH_i$ and $pH_e$ of CaNT murine tumour measured by $^{31}$P MRS. $^{31}$P MR spectra were obtained with an interleaved ISIS localisation acquired with the transmitter frequency set on α-NTP in the upper spectrum (**a**) and on 3-APP in the lower spectrum (**b**) 30 min after the mice were injected with 3-APP. $pH_i$ was measured from the chemical shift difference between α-NTP and $P_i$ in the upper spectrum. $pH_e$ was measured from the chemical shift difference between 3-APP in the lower spectrum and α-NTP in the upper spectrum to correct for the chemical shift artefact (for further details see text).

**Figure 4** (**a**) Time course of $pH_i$ (□) and $pH_e$ (○) measured before and after vascular occlusion with mouse core temperature maintained at room temperature (22–24°C). Symbols represent mean ± s.e.m. ($n = 3$). (**b**) Time course of $pH_i$ (□) and $pH_e$ (○) measured before and after vascular occlusion with mouse core temperature maintained at 33–35°C. Symbols represent mean ± s.e.m. ($n = 3$). *Indicates significant difference ($P<0.05$) from values observed at 0 min.

were treated with FAA, five showed no significant decrease in the NTP/$P_i$ ratio 2 h after treatment whereas in the other four tumours the NTP/$P_i$ ratio started to decrease after 68 min and was significantly different from the control values after 100 min of treatment. In the group of four tumours, the $pH_i$ correspondingly decreased (but not significantly) from 7.08 ± 0.02 to 6.85 ± 0.05 and the $pH_e$ from 6.66 ± 0.08 to 6.23 ± 0.16 by the end of the experiment. In contrast, in the non-responders, $pH_i$ (7.24 ± 0.1 at the beginning of the experiment and 7.16 ± 0.05 at the end) was virtually unchanged as was $pH_e$ (6.75 ± 0.05 at the beginning and 6.59 ± 0.05 at the end).

The decreases in the responders were not significant but in the same direction and of a similar magnitude to those found for total vascular occlusion (see Figure 4). This is probably because FAA takes a long time to exert its effect (relative tumour perfusion ~21% of control after 200 mg $kg^{-1}$; see Hill *et al.*, 1989). However it is not possible to look at later times in these experiments because the 3-APP has to be given before the FAA, when the vasculature is still patent. The 3-APP gradually disappears from the tumour with time leaving no marker of extracellular space at the later times and the signal/noise deteriorates.

**Figure 5** Time course of γ-NTP/$P_i$ ratios measured before and after vascular occlusion with mouse core temperature maintained at either 22–24°C (□) or 33–35°C (○). ($n = 3$) for each point at each temperature. Symbols represent mean ± s.e.m. *Indicates significant difference ($P<0.05$) from values observed at 0 min.

### Discussion

Direct non-invasive measurements of $pH_i$ in tumours have changed the view that existed for many years after Warburg's experiments of the 1920s, namely that tumours were 'acidic' (for review see Griffiths, 1991). Now it is possible to make direct, non-invasive (except for injecting the marker, 3-APP) measurements of $pH_e$. However these measurements do represent volume-average pH values and this may not be ideal in tumours that are known to be histologically heterogeneous. However, some strength lies in using volume averages, especially since they are directly comparable with subsequent metabolic assays done on freeze-clamped tumours which also represent volume averages. One drawback of the volume-average method is that $pH_e$ values obtained by $^{31}$P MRS are likely to be different from $pH_e$ values obtained by

**Table I** Effect of vascular occlusion for 2 h on adenine nucleotides [ATP]/[ADP][$P_i$] and lactate in extracts of CaNT tumours

| *Treatment* | *Total adenine nucleotides* ($\mu mol\, g^{-1}$) | *[ATP]/[ADP][$P_i$]* $M^{-1}$ | *Lactate* ($\mu mol\, g^{-1}$) |
|---|---|---|---|
| Control ($n = 3$) | 2.16 ± 0.14 | 1107 ± 268 | 7.8 ± 0.6 |
| Occlusion ($n = 5$) at 22–24°C | 1.2 ± 0.08* | 51 ± 8.2* | 21 ± 2.3* |
| Occlusion ($n = 7$) at 33–35°C | 0.99 ± 0.17* | 80 ± 31* | 19 ± 2.2* |

*Significantly different from control value ($P<0.05$).

**Figure 6** Representative $^{31}P$ spectra showing preocclusion (**a**) and the decrease in γ-NTP/$P_i$ with time 16–32 min (**b**) and 32–48 min (**c**) post occlusion. Mouse core temperature was maintained at (33–35°C).

**Figure 7** Time course of γ-NTP/$P_i$ ratios measured after treatment with 200 mg $kg^{-1}$ of FAA. (○) are the responders ($n = 4$) and (□) are non-responders ($n = 5$). The 0 min value was obtained from control animals with no treatment ($n = 3$). Symbols represent mean ± s.e.m. *Indicates significant difference ($P<0.05$) from values observed at 36 min, the first post-FAA treatment value obtained.

microelectrodes since microelectrode measurements are invasive and can only sample a few microenvironments within a tumour. The values found in untreated tumours for $pH_e$ by $^{31}P$ MRS were somewhat lower than those found by microelectrode studies in the same tumour type (although performed on different batches at different times (see Parkins *et al.*, 1994*a*), and this may reflect differences between the techniques. The wide range of pH (5.8–7.52) found in tumours by microelectrode measurements (see Wike-Hooley *et al.*, 1984) suggests that they are sampling a mixture of compartments (see also Vaupel *et al.*, 1989) whereas the range of $pH_{APP}$ is very narrow (6.5–6.8) in untreated tumours (see Gillies *et al.*, 1994 and this paper).

In spite of these discrepancies both microelectrodes and 3-APP methods confirm that the extracellular compartment is more acid than the intracellular compartment (see also Vaupel *et al.*, 1989; Griffiths, 1991; Stubbs *et al.*, 1994). Further studies, in which $pH_e$ will be measured on the same tumour by both $^{31}P$ MRS-3-APP and microelectrodes are planned to begin shortly and we hope these studies will resolve the differences between the two methods.

In this study, ISIS localisation of the tumours was chosen to ensure that the intense PCr signal from the body wall did not contribute to the tumour spectra (Howe *et al.*, 1992). However, because the chemical shift difference between 3-APP and α-NTP is >30 p.p.m. there would be a significant chemical shift artefact (Howe *et al.*, 1992). The consequence of this would be that the volumes from which the 3-APP and the α-NTP signals were obtained would be displaced by 2 mm (in three dimensions). In a 0.8 $cm^3$ volume of interest (chosen for most of our experiments), the volumes from which the signals arose would have overlapped by only 43%. To ensure that the chemical shift artefact was minimised and that α-NTP and 3-APP signals came from an identical volume, the double interleaved ISIS acquisition (Maxwell *et al.*, 1994) was used for all ISIS experiments. It should be noted that the chemical shift artefact is negligible when calculating $pH_i$ because the chemical shift difference between $P_i$ and α-NTP is <10 p.p.m.

In theory, absolute concentrations can be obtained from $^{31}P$ MRS since the area under the signals in the MR spectrum are directly proportional to the concentration of the metabolites. However, since ISIS localisation was used, the results presented here have been expressed as peak ratios (i.e. NTP/$P_i$) since absolute sensitivity depends on coil loading and the relative position of the selected voxel to the coil. It should be noted that this ratio (NTP/$P_i$) only approximates the energy status of the tumour in real time. In order to obtain information about the metabolic status of the tumour that might approximate thermodynamic information, [ADP] would have to be ascertained. While [ADP] cannot be determined directly from *in vivo* $^{31}$P MR spectra, total [ADP] (free + bound) can be ascertained by freeze clamping and making extracts of the tumour with subsequent assays of ATP, ADP and $P_i$. Although this does not give true thermodynamic information, it does give the direction, and some indication of the magnitude of the changes observed.

An additional caveat is that the MR spectrum includes all the nucleotide phosphates under the peaks which accounts for the conventional label of NTP, although the major proportion of this peak is ATP (Stubbs *et al.*, 1989). However, the extracts provide data on ATP since HPLC separates ATP from other trinucleotides. The maintenance of adenine nucleotides in tumours compared with control tissue (e.g. liver or kidney) over relatively long periods of ischaemia is supported by anaerobic glycolysis (Williamson *et al.*, 1970; Weber *et al.*, 1971) and the expected large increase in lactate and fall in [ATP]/[ADP][$P_i$] was observed in these tumours.

Lowering $pH_e$ in isolated cell experiments enhances cytotoxicity of certain anti-cancer drugs e.g. doxorubicin, melphalan (for review see Wike-Hooley *et al.*, 1984). Similarly agents that cause vascular occlusion (e.g. FAA), subsequently increase the cytotoxic effect of drugs (such as mitomycin C and chlorambucil *in vivo* (Parkins *et al.*, 1993, 1994*b*). Now that it is possible to monitor $pH_i$ and $pH_e$ simultaneously, we have investigated the effect of total vascular occlusion on $pH_i$ and $pH_e$ at room temperature (22–24°C) and at 33–35°C. The values for $pH_e$ of CaNT tumours were up to 0.4 pH units more acid than $pH_i$. This in itself would enable a drug which was a weak acid (eg. chlorambucil), to partition more favourably into the more alkaline intracellular compartment. However the energy of ATP hydrolysis [ATP]/[ADP][$P_i$] required to maintain the proton gradient is decreased in tumours compared with normal tissues (Stubbs *et al.*, 1994) and after vascular occlusion, this ratio ([ADT]/[ADP][$P_i$]) decreases even further. This combined with the inability of the tumour to remove products of ischaemia such as lactate, contributes to the collapse of ΔpH, caused largely by a decrease in $pH_i$. In the tumours which respond to FAA there is also a decrease, to about the same extent, in both $pH_i$ and $pH_e$.

These results clearly indicate that tumour regions subjected to ischaemia do not display an enhanced differential between $pH_i$ and $pH_e$. Thus, for chemotherapeutic drugs with weak acid functions such as chlorambucil, whose increased activity at reduced $pH_e$ *in vitro* is primarily due to increased uptake as a result of the larger transmembrane ΔpH, no enhanced toxicity in such regions should be expected. However, as mentioned earlier, the tumour cytotoxicity of chlorambucil is enhanced if combined with agents which induce tumour ischaemia such as FAA (Parkins *et al.*, 1994*b*). Two possible explanations for this result are: (i) if chlorambucil is given before an agent which induces tumour ischaemia (i.e. when the vasculature is still patent), then the pharmacokinetics of the agent could be altered by 'trapping' of the drug in the tumour; (ii) it is known that with FAA a small percentage of vessels remain functional and these supply the tumour cells which elicit tumour regrowth following FAA treatment alone. Thus if the majority of the tumour is made ischaemic, acidic metabolites could diffuse and reduce the $pH_e$ in such areas, although, because they still have an adequate blood supply, the cells could still maintain their $pH_i$. In this scenario chlorambucil would have increased toxicity against this remaining subpopulation.

The data obtained show that there are two different responses to FAA – a significant decrease in NTP/$P_i$ ratio and a non-significant decrease in $pH_i$ or $pH_e$ for responders compared to a non-significant decrease in NTP/$P_i$ ratio and no change in $pH_i$ or $pH_e$ for non-responders after FAA treatment. However, this interpretation should be taken with caution because it is possible that the results do not indicate two completely separate responses, but rather the two extremes of the response within a continuum.

Simultaneous and non-invasive monitoring of volume average $pH_i$ and $pH_e$ of solid tumours *in vivo*, demonstrates that $pH_i$ is more alkaline than $pH_e$ and that both decrease after vascular occlusion, or in response to FAA, concomitant with a decrease in the [ATP]/[ADP][$P_i$].

**Acknowledgements**
The authors would like to thank Dr L Bashford for use of the non-parametric 'Robust' computer program for calculating the acid and base points and the p$K_a$ for the pH *vs* chemical shift data and Dr F Howe for helpful discussions. This work was supported by the Cancer Research Campaign Grant No. SP1971/0402.

### References

BERGMEYER HU. (1974). *Methods of Enzymatic Analysis*, 2nd edn, p. 1464. Verlag Chemie: Weinheim.

CHANDRA RAJAN J AND KLEIN L. (1976). Determination of inorganic phosphorus in the presence of organic phosphorus and high concentrations of proteins. *Anal. Biochem.*, **72**, 407–412.

EVELHOCH JL. (1992). The pH of human tumours: facts and problems. In *Radiation Research, a Twentieth Century Perspective*, Vol. 2, Dewey WC, Edington M, Fry, RJM, Hall EJ and Whitmore GF (eds). Academic Press: San Diego, pp 778–784.

EVELHOCH JL. SAPARETO SA, JICK DEL AND ACKERMAN JJH. (1984). *In vivo* metabolic effects of hyperglycemia in murine RIF tumor: a $^{31}$P NMR investigation. *Proc. Natl Acad. Sci. USA*, **81**, 6496–6500.

EVELHOCH JL, BISSERY MC, CHABOT GG, SIMPSON NE, MCCOY CL, HEILBRUN LK AND CORBETT TH. (1988). Flavone acetic acid (NSC 347512)-induced modulation of murine tumor physiology monitored by *in vivo* nuclear magnetic resonance spectroscopy. *Cancer Res.*, **48**, 4749–4755.

GERWECK LE, RHEE JG, KOUTCHER JA, SONG CW AND URANO M. (1991). Regulation of pH in murine tumor and muscle. *Radiat. Res.*, **126**, 206–209.

GILLIES RJ, LIU Z AND BHUJWALLA Z. (1994). $^{31}$P-MRS measurements of extracellular pH of tumors using 3-aminopropylphosphonate. (Cell Physiol. 36) *Am. J. Physiol.*, **267**, C195–C203.

GRIFFITHS JR. (1991). Are cancer cells acidic? (editorial; Review). *Br. J. Cancer*, **64**, 425–427.

HILLS SA, WILLIAMS KB AND DENEKAMP JD. (1989). Vascular collapse after flavone acetic acid: a potential mechanism of its anti-tumour action. *Eur. J. Cancer Clin. Oncol.*, **25**, 1419–1424.

HIRAOKA MA AND HAHN GM. (1989). Comparison between tumor pH and cell sensitivity to heat in RIF-1 tumors. *Cancer Res.*, **49**, 3734–3736.

HOWE FA, STUBBS M, RODRIGUES LM AND GRIFFITHS JR. (1992). An assessment of artefacts in localised and non-localised $^{31}$P MRS studies of phosphate metabolites and pH in rat tumours. *NMR Biomed.*, **6**, 43–52.

HWANG YC, KIM S-G, EVELHOCH JL, SEYEDSADR M AND ACKERMAN JJ. (1991). Modulation of murine radiation-induced fibrosarcoma-1 tumor metabolism and blood flow *in situ* via glucose and mannitol administration monitored by $^{31}$P and $^{2}$H nuclear magnetic resonance spectroscopy. *Cancer Res.*, **51**, 3108–3118.

JAHDE E, VOLK T, ATEMA A, SMETS LA, GLUSENKAMP KH AND RAJEWSKY MF. (1992). pH in human tumor xenografts and transplanted rat tumors: effect of insulin, inorganic phosphate, and m-iodobenzylguanidine. *Cancer Res.*, **52**, 6209–6215.

LOWRY OH AND LOPEZ JA. (1946). The determination of inorganic phosphate in the presence of labile phosphate esters. *J. Biol. Chem.*, **162**, 421–428.

MAIDORN RP, CRAGOE EJJ AND TANNOCK IF. (1993). Therapeutic potential of analogues of amiloride: inhibition of the regulation of intracellular pH as a possible mechanism of tumour selective therapy. *Br. J. Cancer*, **67**, 297–303.

MAXWELL RJ, MCCOY CL AND GRIFFITHS JR. (1994). Assessment of double volume acquisition (DIVA) method to minimise chemical shift artifacts in localised $^{31}P$ MRS (abstract). *Soc. Magn. Reson.*, **3**, 1337.

MURRAY JC, SMITH KA AND THURSTON G. (1989). Flavone acetic acid induces a coagulopathy in mice. *Br. J. Cancer*, **60**, 729–733.

NEGENDANK W. (1992). Studies of human tumours by MRS: a review. *NMR Biomed.*, **5**, 303–324.

NEWELL KW, WOOD P, STRATFORD I AND TANNOCK I. (1992). Effects of agents which inhibit the regulation of intracellular pH on murine solid tumours. *Br. J. Cancer*, **66**, 311–317.

ORDIDGE RJ, CONNELLY A AND LOHMAN JAB. (1986). Image-selected *in vivo* spectroscopy (ISIS). A new technique for spatially selective NMR spectroscopy. *J. Magn. Reson.*, **66**, 283–294.

PARKINS CS, HILL SA, LONERGAN SJ, HORSMAN MR, CHADWICK JA AND CHAPLIN DJ. (1994*a*). Ischaemia-induced cell death in tumors: importance of temperature and pH. *Int. J. Radiat. Oncol. Biol. Phys.*, **29**, 499–503.

PARKINS CS, CHADWICK JA AND CHAPLIN DJ. (1994*b*). Enhancement of chlorambucil cytotoxicity by combination with flavone acetic acid in a murine tumour. *Anticancer Res.*, **14**, 1603–1608.

PARKINS CS, DENEKAMP JD AND CHAPLIN JD. (1993). Enhancement of mitomycin-C cytotoxicity by combination with flavone acetic acid in a murine tumour. *Anticancer Res.*, **13**, 1437–1442.

PRICHARD JW, ALGER JR, BEHAR KL, PETROFF OA AND SHULMAN RG. (1983). Cerebral metabolic studies *in vivo* by $^{31}P$ NMR. *Proc. Natl Acad. Sci. USA*, **80**, 2748–2751.

STUBBS M, RODRIGUES LM, HOWE FA, WANG J, JEONG KS, VEECH RL AND GRIFFITHS JR. (1994). The metabolic consequences of a reversed pH gradient in rat tumours. *Cancer Res.*, **54**, 4011–4016.

STUBBS M, BHUJWALLA ZM, TOZER GM, RODRIGUES LM, MAXWELL RJ, MORGAN R, HOWE FA AND GRIFFITHS JR. (1992). An assessment of $^{31}P$ MRS as a method of measuring pH in rat tumours. *NMR Biomed.*, **5**, 351–359.

STUBBS M, RODRIGUES LM AND GRIFFITHS JR. (1989). Growth studies of subcutaneous rat tumours: comparison of $^{31}P$ NMR spectroscopy, acid extracts and histology. *Br. J. Cancer*, **60**, 701–707.

TANNOCK IF AND ROTIN D. (1989). Acid pH in tumors and its potential for therapeutic exploitation. *Cancer Res.*, **49**, 4373–4383.

VAN DER VEEN JWC, DE BEER R, LUYTEN PR AND VAN ORMONDT D. (1988). Accurate quantification of *in vivo* $^{31}P$ NMR signals using the variable projection method and prior knowledge. *Magn. Reson. Med.*, **6**, 92–98.

VAUPEL P, KALLINOWSKI F AND OKUNIEFF P. (1989). Blood flow, oxygen and nutrient supply, and metabolic microenvironment of human tumors: a review. *Cancer Res.*, **49**, 6449–6465.

VORHEES WD AND BABBS CF. (1982). Hydralazine-enhanced selective heating of transmissible venereal tumour implants in dogs. *Eur. J. Cancer*, **18**, 1027–1033.

WEBER G, STUBBS M AND MORRIS HP. (1971). Metabolism of hepatomas of different growth rates *in situ* and during ischaemia. *Cancer Res.*, **31**, 2177–2183.

WIKE-HOOLEY JL, HAVEMAN J AND REINHOLD HS. (1984). The relevance of tumour pH to the treatment of malignant disease. *Radiother. Oncol.*, **2**, 343–366.

WILLIAMSON DH, KREBS HA, STUBBS M, PAGE MA, MORRIS HP AND WEBER G. (1970). Metabolism of renal tumors *in situ* and during ischemia. *Cancer Res.*, **30**, 2049–2054.